# DetectFormer: Category-Assisted Transformer for Traffic Scene Object Detection

**DOI:** 10.3390/s22134833

**Published:** 2022-06-26

**Authors:** Tianjiao Liang, Hong Bao, Weiguo Pan, Xinyue Fan, Han Li

**Affiliations:** 1 Beijing Key Laboratory of Information Service Engineering, Beijing Union University, Beijing 100101, China; 20201083510925@buu.edu.cn (T.L.); baohong@buu.edu.cn (H.B.); 20201083510910@buu.edu.cn (X.F.); 20201083510918@buu.edu.cn (H.L.); 2 College of Robotics, Beijing Union University, Beijing 100101, China

**Keywords:** autonomous driving, deep learning, object detection, transformer

## Abstract

Object detection plays a vital role in autonomous driving systems, and the accurate detection of surrounding objects can ensure the safe driving of vehicles. This paper proposes a category-assisted transformer object detector called DetectFormer for autonomous driving. The proposed object detector can achieve better accuracy compared with the baseline. Specifically, ClassDecoder is assisted by proposal categories and global information from the Global Extract Encoder (GEE) to improve the category sensitivity and detection performance. This fits the distribution of object categories in specific scene backgrounds and the connection between objects and the image context. Data augmentation is used to improve robustness and attention mechanism added in backbone network to extract channel-wise spatial features and direction information. The results obtained by benchmark experiment reveal that the proposed method can achieve higher real-time detection performance in traffic scenes compared with RetinaNet and FCOS. The proposed method achieved a detection performance of 97.6% and 91.4% in AP50 and AP75 on the BCTSDB dataset, respectively.

## 1. Introduction

Vision-based object detection in traffic scenes plays a crucial role in autonomous driving systems. With the rapid development of autonomous driving, the performance of object detection has made significant progress. The traffic object (e.g., traffic signs, vehicles, and pedestrians) can be detected automatically by extracting the features. The result of perceiving the traffic scenario can ensure the safety of the autonomous vehicle. This kind of method can be divided into anchor-based and anchor-free.

Deep-learning-based object detection can be divided into single-stage and multi-stage object detection. The multi-stage algorithms extract the region of interest first, and then the location of the object is determined in these candidate areas. The single-stage algorithm’s output the location and category with dense bounding boxes directly on the original image. These detection algorithms classify each anchor box or key point and detect different categories independently, while ignoring the relationships between categories. There exists a specific relationship between other objects, such as probability, location, and scale of different objects in a particular environment, which is essential for object detection and can improve object detection accuracy.

This relationship between categories exists in many cases in traffic scenarios. For example, pedestrians appearing in highway scenes and vehicles appearing on the pedestrian path are low-probability events, which indicates the connection between object categories and scenarios. Secondly, the signs “Passing” and “No Passing” should not appear in the same scene, which indicates the connection between different object categories. There exist specific implicit relationships between object categories and the background of traffic scenes. Existing object detection methods do not consider this relationship in scenes, and their classification subnetwork is trained to independently classify different objects as individuals without the objects knowing each other, which results in the model underperforming in terms of fitting the distribution of objects and the scene background. Additionally, the model does not thoroughly learn the features required by the detection task and will cause a gap in the classification confidence between categories, which influences the detection performance.

Based on the above-mentioned assumptions, this paper proposes a category-assisted transformer object detector to learn the relationships between different objects called DetectFormer, based on the single-stage method. The motivation of this study was to allow the classification subnetwork to fit better the distribution of object categories with specific scene backgrounds and ensure that the network model is more focused on this relationship.

Transformer [1] is widely used in natural language processing, machine translation, and computer vision because of its ability to perceive global information. Specifically, the vision transformer (ViT) [2] and DETR [3] have been proposed and applied to computer vision. Previous studies have used transformers to capture global feature information and reallocate network attention to features, which is called self-attention. In this study, DetectFormer was built based on the transformer concept. Still the inputs and structure of the multi-head attention mechanism are different because the purpose of DetectFormer is to improve the detection accuracy with the assistance of category information.

The contributions of this study are as follows:

(1) The Global Extract Encoder (GEE) is proposed to extract the global information of the image features output by the backbone network, enhancing the model’s global perception ability.
(2) A novel category-assisted transformer called ClassDecoder is proposed. It can learn the object category relationships and improve the model’s sensitivity by implicitly learning the relationships between objects.
(3) The attention mechanism is added to the backbone network to capture cross-channel, direction-aware and position-sensitive information during feature extraction.
(4) Efficient data augmentation methods are proposed to enhance the diversity of the dataset and improve the robustness of model detection.

The rest of this paper is organized as follows. In Section 2, we introduce object detection algorithms and transformer structure. Details of the proposed DetectFormer are presented in Section 3. In Section 4, the model’s implementation is discussed, and the model is compared with previous methods. The conclusions and direction of future work are discussed in Section 5.

## 2. Related Work

### 2.1. Object Detection

Traditional object detection uses HOG [4] or DPM [5] to extract the image features, and then feed them into a classifier such as SVM [6]. Chen et al. [7] use SVM for traffic light detection. In recent years, deep learning based object detection algorithms have achieved better performance in terms of accuracy compared with traditional methods and have become a research hotspot. Generally, there are two types of object detection based on deep convolutional networks: (1) multi-stage detection, such as R-CNN series [8,9,10], and Cascade R-CNN [11]; (2) one-stage detection, which is also known as the dense detector and can be divided into anchor-based methods (for example, the You Only Look Once series [12,13,14] and RetinaNet [15]) and anchor-free methods (for example, FCOS [16], CenterNet [17], and CornerNet [18]). Multi-stage detection methods extract features of the foreground area using region proposal algorithms from preset dense candidates in the first stage. The bounding boxes of objects are regressed in the subsequent steps. The limitation of this structure is that it reduces the detection speed and cannot satisfy the real-time requirements of autonomous driving tasks. Single-stage detection methods directly detect the object and regress the bounding boxes different from multi-stage methods, which can avoid the repeated calculation of the feature map and obtains the anchor boxes directly on the feature map. He et al. [19] proposed a detection method using CapsNet [20] based on visual inspection of traffic scenes. Li et al. [21] proposed improved Faster R-CNN for multi-object detection in a complex traffic environments. Lian et al. [22] proposed attention fusion for small traffic object detection. Liang et al. [23] proposed a light-weight anchor-free detector for traffic scene object detection. However, their models cannot capture global information limited by the size of the receptive field. The above-mentioned approaches obtain local information when extracting image features, and enlarge the receptive field by increasing the size of the convolution kernel or stacking the number of convolution layers. In recent years, transformers have been introduced as new attention-based building blocks applied to computer vision, they have achieved superior performance because they can obtain the global information of the image without increasing the receptive field.

### 2.2. Transformers Structure

The transformer is a new encoder–decoder architecture introduced by Vaswani et al. [1] first used in machine translation and has better performance than LSTM [24], GRU [25], RNNs [26] (MoE [27], GNMT [28]) in translation tasks. Transformer extracts features by aggregating global information, making it suited for long sequence prediction tasks and other information-heavy tasks, which has better performance than other RNN-based models in natural language processing [29,30], speech processing [31], transfer learning [32]. It is comparable to the performance of CNN in computer vision as a new framework. Alexey et al. [2] proposed a vision transformer, which applied a transformer to computer vision and image classification tasks. Nicolas et al. [3] proposed DETR, which applied a transformer to object detection task. Yan et al. use a transformer to predict long-term traffic flow [33]. Cai et al. [34] use a transformer to capture the spatial dependency for continuity and periodicity time series.

Although the transformer structure shows strong performance, the training based on the transformer takes a long time, and requires a large amount of data sets and ideal pre-training. This paper proposes a learnable object relationship module based on a transformer with self-attention, and a single-stage detector was designed to complete the task of traffic scene object detection. Compared with other methods, the proposed method achieves better detection performance in a shorter training time.

## 3. Proposed Method

**Figure 1:**
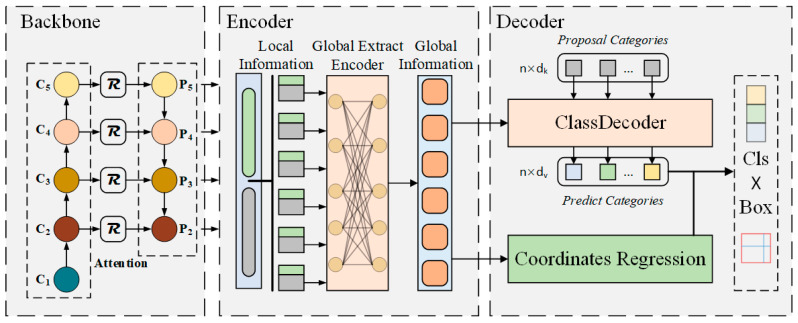
The overall architecture of the proposed method. The architecture can be divided into three parts: backbone, encoder, and decoder. The backbone network is used to extract image features, the encoder is used to enhance the model’s global perception ability, and the decoder is used to detect the objects in traffic scenes.

The overall pipeline of our proposed method is shown in Figure 1. The main contributions of the proposed method are the following three parts: (1) attention mechanism in backbone network based on position information; (2) the Global Extract Encoder can enhance the model’s global perception ability; (3) a novel learnable object relationship module called ClassDecoder. Finally, efficient data augmentation was used to improve the robustness of the model.

### 3.1. Global Extract Encoder

The convolutional neural network is usually affected by the kernel size, network depth, and other factors, causing the receptive field cannot cover the whole area of the image, which is challenging to learn the relationship between long-distant regions or pixels. When extracting the features of the object, the network cannot obtain global information.

**Figure 2:**
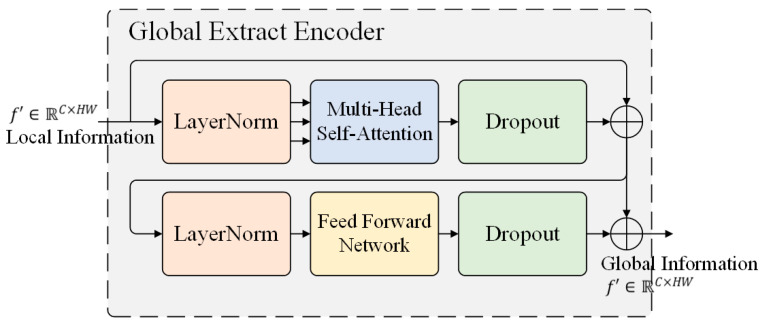
Structure of Global Extract Encoder. The multi-head self-attention learning the global information from feature maps and feedforward network enables Global Extract Encoder to acquire the ability of nonlinear fitting.

Inspired by the transformer architecture and the vision transformer, this study designed the Global Extract Encoder (GEE) to enhance the model’s global perception ability. As shown in Figure 1, the GEE accepts the image features $f\in {\mathbb{R}}^{C\times H\times W}$ extracted from the backbone network, performs global information perception on f, and sends $f^{out}$ to the following Decoder for object detection. The typical values used in this study are C=2048 and $H,W=\frac{H_{I}}{32},\frac{W_{I}}{32}$, where $H_{I},W_{I}$ are the height and width of the original image $x_{in}\in {\mathbb{R}}^{3\times H_{I}\times W_{I}}$. The structure of GEE is shown in Figure 2 and consists of two primary modules. The first module is the multi-head self-attention layer, and the second one is the feedforward network (FFN). Residual connections ⊕ are used between each sub-layer.

We split the feature maps into patches, and collapsed the spatial dimensions of f from ${\mathbb{R}}^{C\times H\times W}$ to a one dimension sequence ${\mathbb{R}}^{C\times HW}$. Then, a fixed position embedding is added to the feature sequence $f^{\prime }\in {\mathbb{R}}^{C\times HW}$ owing to permutation invariance and fed into GEE. The obtained information from different subspaces and positions by adding multi-head self-attention ℋ.

(1) $$W_{i}^{f\left(j\right)}=w^{\left(j\right)}f^{\prime }\;\;\;\;j=1,2,3\;,$$

(2) $$h_{i}=Softmax\left(\frac{W_{i}^{f\left(i\right)}W_{i}^{f\left(j\right)}{}^{T}}{\sqrt{HW/n}}\right)W_{i}^{f\left(k\right)}\;\;\;i\neq j\neq k,$$

(3) $$ℋ=Concat\left(h_{1},h_{2},\ldots ,h_{n}\right)w^{\left(ℋ\right)},$$

where projection matrix $w^{\left(j\right)}\in {\mathbb{R}}^{c\times HW}\;j=1,2,3.$ Additionally, $w^{\left(ℋ\right)}\in {\mathbb{R}}^{nHW\times c}$, and *n* donates the number of heads. The feedforward network (FFN) enables GEE the ability of nonlinear fitting. After global feature extraction, $f^{\prime }$ expands the spatial dimension into C×H×W. Thus, the dimensions of the GEE module output $f^{out}\in {\mathbb{R}}^{C\times H\times W}$ are consistent with the input dimensions, and the model can obtain long distance regional relationships and global information rather than local information when extracting object features.

### 3.2. Class Decoder

**Figure 3:**
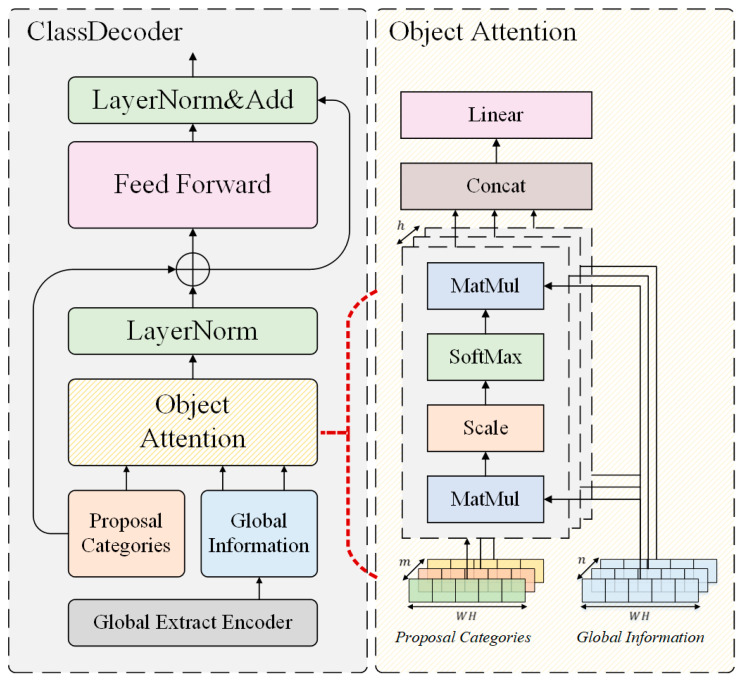
Structure of ClassDecoder. Proposal categories learn the relationship between different categories and classify objects based on Global Information.

To learn the object category relationships and improve the model’s sensitivity to the categories by implicitly learning the relationships between objects, a novel learnable object relationship module called ClassDecoder is proposed. The structure of ClassDecoder is shown in Figure 3 and is similar to the transformer architecture. However, this study disregarded the self-attention mechanism, the core of transformer blocks, and designed a module from the perspective of object categories to implicitly learn the relationship between categories, including the foreground and background. Here, 1 × 1 convolution was used to reduce the channel dimension of the global feature map $f^{out}$ from C to a smaller dimension m, and the spatial dimensions were collapsed to create a new feature sequence $G\in {\mathbb{R}}^{m\times HW}$.

(4) $$G=F\left(\varphi \left(f^{out}\right)\right),$$

where the φ(.) means 1 × 1 convolutional operation to reduce the channel dimension of $f^{out}$, and *F*(.) means collapse operator, which transforms two-dimensional feature matrices into feature sequences.

ClassDecoder block requires two inputs: the feature sequence G and the proposal categories P. The proposed ClassDecoder is to detect different categories of objects, using proposal categories to predict the confidence vector of each category, and the depth n of ClassDecoder represents the number of categories. Then, the convolution operation is used to generate the global descriptor of each vector. Finally, the softmax function is used to output the prediction result of the category.

(5) $$f^{p}=Softmax\left(\frac{GP^{T}}{\sqrt{d_{k}}}\right)P,$$

(6) $$y_{class}=Softmax\left(\sigma \left(\varphi (f^{p}\right)\right)).$$

where the global information *G* ($G\in {\mathbb{R}}^{n\times d_{k}}$), the proposal categories *P* ($P\in {\mathbb{R}}^{m\times d_{v}}$), and m is the same as the first dimension of G. In this study, the dimensions of $d_{k}$ and $d_{v}$ were set to be the same and equal to the feature channels H×W; P denotes various learnable sequences that are referred to as proposal categories and are independently decoded into class labels, resulting in n final class predictions, where n denotes the total number of dataset categories in anchor-free methods and is the product of the number of categories and number of anchor boxes in anchor-based methods.

There are many ways to initialize the proposal categories. Transformer architecture does not contain any inductive bias; this study attempted to feed prior knowledge into ClassDecoder, and proposal categories were initialized as follows. A 1 × 1 convolution was used to reduce the dimension of g and reduce the original m dimension to the n dimension (generally, n≪m), where n represents the total number of categories in the dataset of the detection task based on the anchor-free method. ClassDecoder globally reasons about all categories simultaneously using the pair-wise relationships between objects while learning the relationship between categories, including the foreground and background.

### 3.3. Attention Mechanism in the Backbone Network

**Figure 4:**
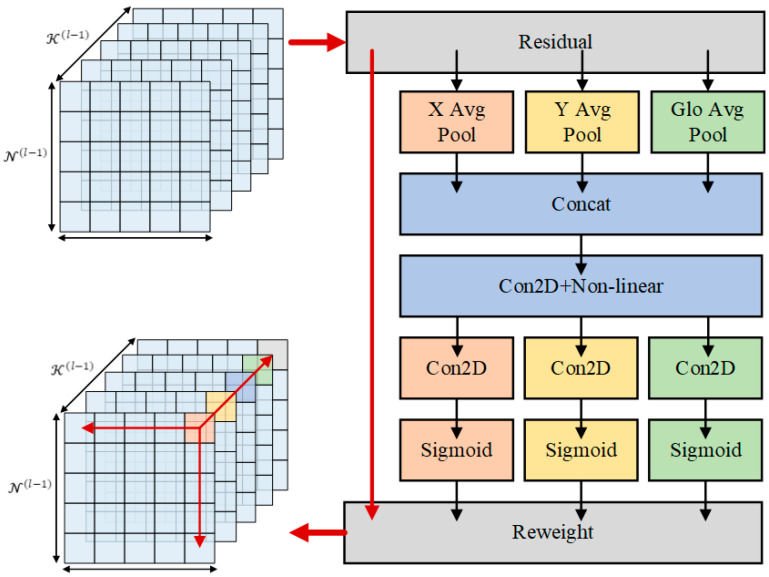
The attention mechanism in backbone network. We propose the global encoding for channel-wise spatial information and extract X and Y direction information for the location attention features.

The attention mechanisms in computer vision can enhance the objects in the feature maps. CBAM [35] attempts to utilize position information by reducing the channel dimension of the input tensor and using convolution to compute spatial attention. Different from CBAM, our proposed method adds a location attention feature to build the direction-aware information, which can improve the network more accurately locate objects, by capturing precise location information in two different spatial directions. A global encoding for channel-wise spatial information is added based on Coordinate Attention [36]. Specifically, the features $x_{c}\left(i,j\right)$ are aggregated along W and H spatial directions to obtain feature maps of perception in two directions. These two features $z_{c}^{h}\left(h\right)$ and $z_{c}^{w}\left(h\right)$ allow the attention module to obtain long-term dependencies along with different spatial directions. The concatenate operation *F* is performed with the channel descriptor $z_{c}^{g}$ with global spatial information. Then, the convolution function φ is used to transform them and obtain the output 𝒫, as shown in Figure 4.

$z_{c}^{g}$, $z_{c}^{h}\left(h\right)$ and $z_{c}^{w}\left(h\right)$ are defined as follows:

(7) $$z_{c}^{g}=\frac{1}{H\times W}\sum_{i=1}^{H}\sum_{j=1}^{W}x_{c}\left(i,j\right),$$

(8) $$z_{c}^{h}\left(h\right)=\frac{1}{W}\sum_{0\leq i<W}x_{c}\left(h,i\right),$$

(9) $$z_{c}^{w}\left(w\right)=\frac{1}{H}\sum_{0\leq j<H}x_{c}\left(j,w\right),$$

(10) $$𝒫=\varphi \left(F\left[z_{c}^{g},z_{c}^{h}\left(h\right),z_{c}^{w}\left(w\right)\right]\right).$$

where $x_{c}$ is the input from the features extracted from the previous layer associated with the c-channel, φ(.) is the convolutional operation, and F[.] is concatenate operation. After the output of different information 𝒫 through their respective convolution layer (.), the normalization is activated by sigmoid activation function σ(.). The final output $y_{c}$ is the multiply of the original feature map and information weights.

(11) $$f^{w}=\sigma \left(\varphi_{w}\left({𝒫}^{w}\right)\right),$$

(12) $$f^{h}=\sigma \left(\varphi_{h}\left({𝒫}^{h}\right)\right),$$

(13) $$f^{g}=\sigma \left(\varphi_{g}\left({𝒫}^{g}\right)\right),$$

(14) $$y_{c}\left(i,j\right)=x_{c}\left(i,j\right)\times f_{c}^{w}\left(j\right)\times f_{c}^{h}\left(i\right)\times f_{c}^{g}\left(i,j\right).$$

The proposed attention mechanism in the backbone could be applied to different kinds of networks. As shown in the following experimental part, the improved attention mechanism can be plugged into lightweight backbone networks and improve the network detection capability.

### 3.4. Data Augmentation

**Figure 5:**
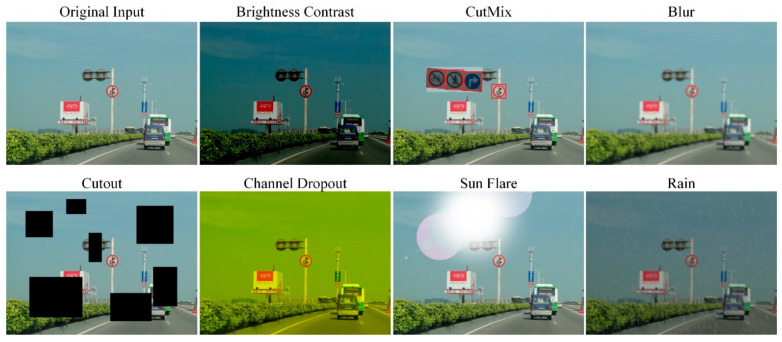
Efficient data augmentation for traffic scene images. Different augmentation methods are used to simulate the complex environment.

Traffic scene object detection is usually affected by light, weather, and other factors. The data-driven deep neural networks require a large number of labeled images to train the model. Most traffic scene datasets cannot cover all complex environmental conditions. In this paper, we use three types of data augmentation methods global pixel level, spatial level, and object level, as shown in Figure 5. Specifically, we use Brightness Contrast, Blur, and Channel Dropout for illumination transformation; we use Rain, Sun Flare, and Cutout [37] for the spatial level data augmentation, Mixup, CutMix [38] for the object level augmentation. The data augmented by these methods can simulate complex traffic scenarios, which can improve the detection robustness of the model.

## 4. Experience and Results

### 4.1. Evaluation Metrics

The average precision (AP) metrics were used to evaluate the detection performance, including AP at different IoU thresholds (AP, AP_50_, AP_75_) and AP for different scale objects (AP_S_, AP_M_, AP_L_), which consider both recall and precision. The top-n accuracy was used to evaluate the classification ability of different methods. Top-n represents the truth value of the object in the first n confidence results of the model. We also use parameters and FLOPs (floating-point operations per second) to measure the volume and computation of different models.

### 4.2. Datasets

Detection performance in traffic scenes is evaluated using the BCTSDB [39], KITTI [40], and COCO [41] datasets to evaluate the generalization ability. The KITTI dataset contains 7481 training images and 7518 test images, totaling 80,256 labeled objects with three categories (e.g., vehicle, pedestrian, and cyclist). The BCTSDB dataset contains 15,690 traffic sign images, including 25,243 labeled traffic signs. The COCO dataset is used to test the generalization ability of the model including 80 object categories and more than 220 K labeled images.

### 4.3. Implementation and Training Details

The network structure constructed by PyTorch and the default hyperparameters used were the same as those for MMDetection [42] unless otherwise stated. Two NVIDIA TITAN V graphics cards with 24 GB VRAM were used to train the model. The linear warming up policy was used to start the training, where the warm-up ratio was set to 0.1. The optimizer of DetectFormer is AdamW [43]; the initial learning rate is set to 10^−4^, and the weight decay is set to 10^−4^. The backbone network is established using pre-trained weights from ImageNet [44], and other layers used Xavier [45] for parameter initialization except for the proposal categories. The input images are scaled to a full scale of 640 × 640, while maintaining the aspect ratio.

### 4.4. Performances

We first evaluate the effectiveness of the different proposed units. The ClassDecoder head, Global Extract Encoder, Attention, Anchor-free head, and Data augmentation are gradually added to the RetinaNet baseline on COCO and BCTSDB dataset to test the generalization ability of the proposed method and the detection ability in the traffic scene, as shown in Table 1 and Table 2, respectively.

We further compare the different performances of anchor-based and anchor-free methods on KITTI dataset. As shown in Table 3, the detection performance of an anchor-free detector with Feature Pyramid Network (FPN) [46] is better than the anchor-based detector. FPN plays a crucial role in improving detection accuracy based on the anchor-free method.

**Figure 6:**
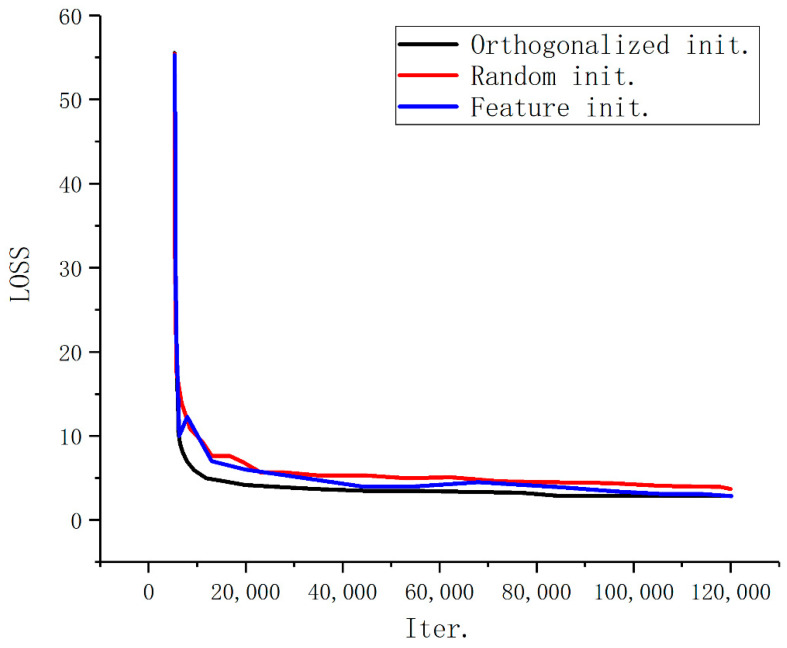
The loss curves for different initialization methods.

For the initialization method of proposal categories, we compare different methods, as shown in Figure 6. The experiment shows that the orthogonalized initial parameter method better than the random initialization method in the early stage of training. The advantage becomes less obvious as the training continue.

The efficiency of attention and detection results of DetectFormer with different number of parameter backbone networks, from light-weight backbone network (MobileNetv3 [47]) to high-performance backbone network (ResNet101 [48]) are shown in Table 4, which shows that it can improve the detection performance of the model by inserting attention mechanism into the backbone network, especially in the lightweight backbone network, our method is competitive in lightweight networks.

Table 5 presents the classification performance of baseline methods and that of the proposed method on the BCTSDB dataset. Anchor-based and anchor-free methods were used to compare RetinaNet and FCOS, respectively. The experimental results reveal that DetectFormer is helpful in improving the classification ability of the model. Remarkably, DetectFormer can reduce the computation and parameter number of the detection networks.

**Figure 7:**
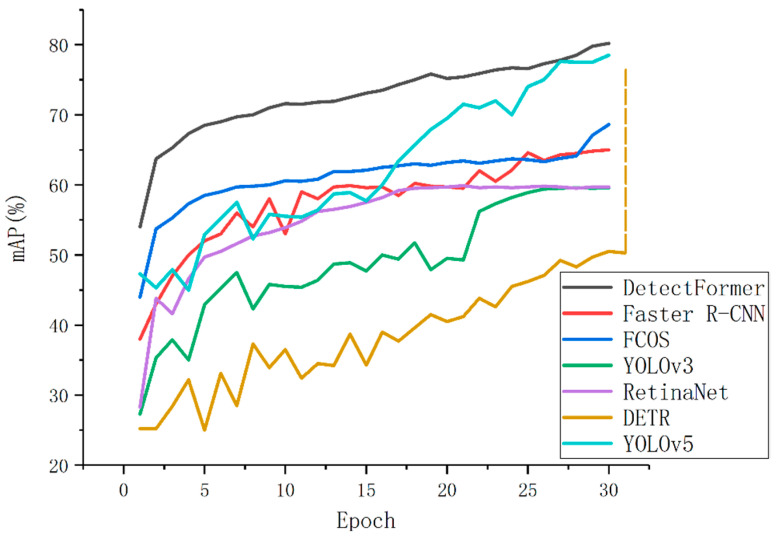
The detection results with different methods on the BCTSDB dataset. Our model can achieve higher detection accuracy in shorter training epochs. In particular, DETR requires more than 200 training epochs for high precision detection.

The convergence curves among the DetectFormer and other SOTA (state-of-the-art) methods, including RetinaNet, DETR, Faster R-CNN, FCOS, and YOLOv5, are shown in Figure 7, which illustrates that DetectFormer achieves better performance with efficient training and accurate detection. The vertical axis is the detection accuracy.

Table 6 shows the detection results on BCTSDB dataset produced by multi-stage methods (e.g., Faster R-CNN, Cascade R-CNN) and single-stage methods, including anchor-based methods (e.g., YOLOv3, RetinaNet) and the anchor-free method FCOS. DetectFormer shows high detection accuracy and more competitive performance. The AP, AP50, and AP75 are 76.1%, 97.6%, and 84.3%, respectively. DetectFormer can suit the distribution of object categories and boost detection confidence in the field of autonomous driving better than other networks.

The proposed method was also evaluated on the KITTI dataset. As shown in Table 7, compared with other methods, DetectFormer shows better detection results.

**Figure 8:**
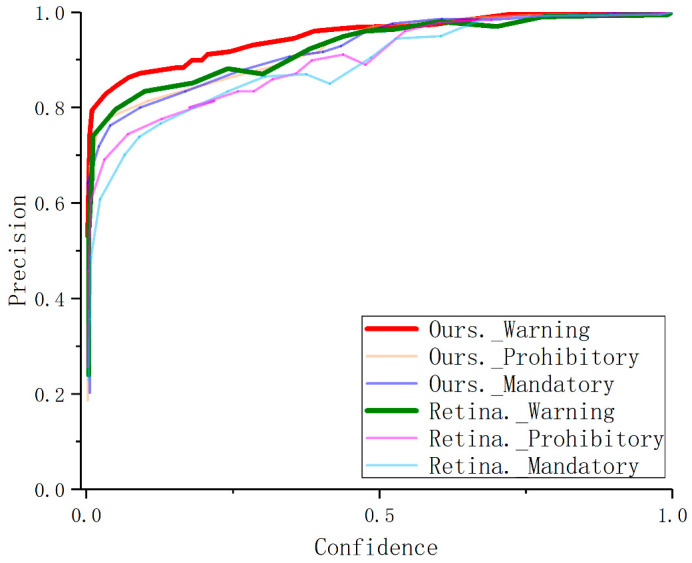
Precision curves of the proposed method and RetinaNet. Our model has high detection accuracy even with low confidence.

**Figure 9:**
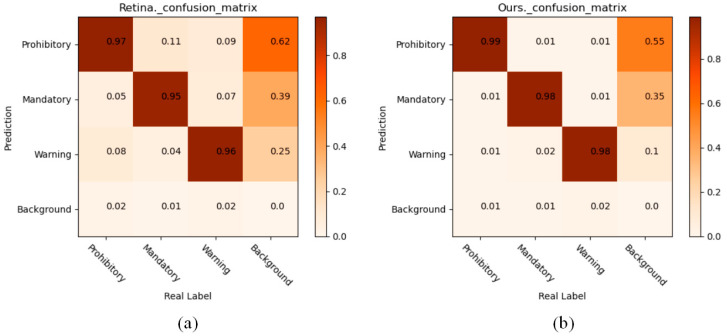
Confusion matrix of the proposed method and RetinaNet. The darker the block, the larger the value it represents. Compared with RetinaNet, the proposed method can obtain more category information and help to classify the objects. (**a**) Confusion matrix of RetinaNet. (**b**) Confusion matrix of DetectFormer.

Figure 8 and Figure 9 and show that DetectFormer can improve the model’s sensitivity to categories by implicitly learning the relationships between objects.

**Figure 10:**
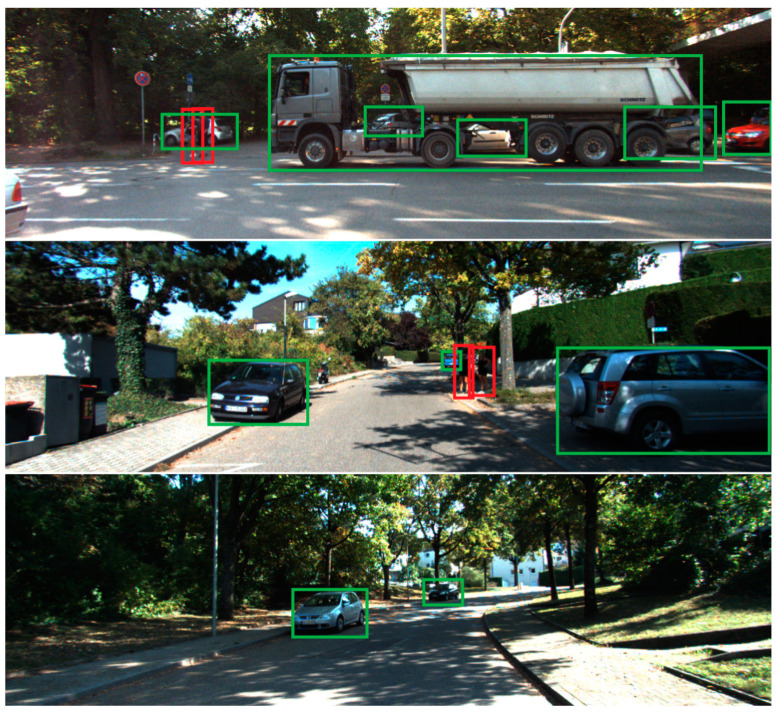
Detection Results on KITTI dataset. Our method can detect different objects in traffic scenes accurately, and even identify overlapping objects and dense objects.

**Figure 11:**
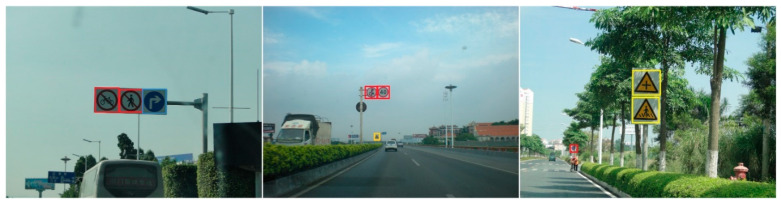
Detection results on BCTSDB dataset. Our method can detect traffic signs at different scales with high precision.

The detection results are shown in Figure 10 and Figure 11 on the KITTI and BCTSDB datasets, respectively. The results demonstrate the proposed method’s effectiveness in traffic scenarios. Three types of traffic signs on the BCTSDB dataset, including warning, prohibitory, mandatory, and three types of traffic objects on the KITTI dataset, including car, pedestrian, cyclist were detected. The detection result does not include other types of traffic objects such as a motorcycle in Figure 10, but the proposed model can detect those kinds of objects.

## 5. Discussion

Why can ClassDecoder improve the classification ability of models? In this paper, we propose ClassDecoder to improve the classification ability, which is designed based on the transformer architecture without any convolution operations. The model interacts with different background feature maps in scaled dot-product attention and multi-head attention by using proposal categories, and learns the implicit relationship between the background and the category by using the key-value pair idea in the Transformer. The number of proposal categories is equal to the number of object categories, and the parameters of proposal categories are learnable. The input of ClassDecoder is the feature maps, and proposal categories, and the output is the prediction category of the current bounding box. The output dimensions are the same as those of the proposal categories, and the proposal categories are associated with the output in the role of Query (Query-Key-Value relationship in transformer architecture). It can be understood that the proposal categories are vectors that can be learned, and their quantity represents the confidence vectors corresponding to different categories of the current bounding box. Then, the model converts the confidence vector into category confidence through feed-forward network. The category with the highest confidence is the category of the predicted bounding box.

## 6. Conclusions

This paper proposes a novel object detector called DetectFormer, which is assisted by a transformer to learn the relationship between objects in traffic scenes. By introducing the GEE and ClassDecoder, this study focused on fitting the distribution of object categories to specific scene backgrounds and implicitly learning the object category relationships to improve the sensitivity of the model to the categories. The results obtained by experiments on the KITTI and BCTSDB datasets reveal that the proposed method can improve the classification ability and achieve outstanding performance in complex traffic scenes. The AP50 and AP75 of the proposed method are 97.6% and 91.4% on BCTSDB, and the average accuracies of car, pedestrian, and cyclist are 86.6%, 79.5%, and 81.7% on KITTI, respectively, which indicates that the proposed method achieves better results compared to other methods. The proposed method improved detection accuracy, but it still encountered many challenges when applied to natural traffic scenarios. The experiment in this paper is trained on public datasets and real traffic scenes facing challenges with complex lighting and weather factors. Our future work is focused on object detection in an open environment and the deployment of models to vehicles.

## Figures and Tables

**Table 1:** The ablation study on the COCO dataset.

Methods	Parameters (M)	FLOPs (G)	AP (%)	AP50 (%)	AP75 (%)
RetinaNet baseline	37.74	95.66	32.5	50.9	34.8
+ClassDecoder	35.03 (−2.71)	70.30 (−25.36)	34.6 (+2.1)	53.5 (+2.6)	36.1 (+1.3)
+Global Extract Encoder	36.95 (+1.92)	90.45 (+20.15)	36.2 (+1.6)	55.7 (+2.2)	37.8 (+1.7)
+Attention	37.45 (+0.5)	90.65 (+0.2)	38.3 (+2.1)	58.3 (+2.6)	39.3 (+1.5)
+Anchor-free	37.31 (−0.14)	89.95 (−0.7)	38.9 (+0.6)	59.1 (+0.8)	39.6 (+0.3)
+Data augmentation	37.31 (+0)	89.95 (+0)	41.3 (+2.4)	61.8 (+2.7)	41.5 (+1.9)

**Table 2:** The ablation study on the BCTSDB dataset.

Methods	Parameters (M)	FLOPs (G)	AP (%)	AP50 (%)	AP75 (%)
RetinaNet baseline	37.74	95.66	59.7	89.4	71.2
+ClassDecoder	35.03 (−2.71)	70.30 (−25.36)	61.6 (+3.7)	91.8 (+2.4)	75.8 (+4.6)
+Global Extract Encoder	36.95 (+1.92)	90.45 (+20.15)	63.4 (+3.4)	93.9 (+2.1)	80.6 (+4.8)
+Attention	37.45 (+0.5)	90.65 (+0.2)	65.2 (+3.1)	95.1 (+1.2)	84.2 (+3.6)
+Anchor-free	37.31 (−0.14)	89.95 (−0.7)	65.8 (+2.1)	95.7 (+0.6)	87.4 (+3.2)
+Data augmentation	37.31 (+0)	89.95 (+0)	76.1 (+4.1)	97.6 (+1.9)	91.4 (+4.0)

**Table 3:** Comparison of anchor-based and anchor-free methods on the KITTI dataset.

Methods	Detector	Car (%)	Pedestrian (%)	Cyclist (%)
DetectFormer	Anchor-based	83.24	70.11	73.54
	Anchor-free	69.45	61.15	62.24
	Anchor-free w/. FPN	86.59	79.45	81.71

**Table 4:** The performance of attentional mechanism in different backbone networks on BCTSDB dataset.

Backbone	Params.	FLOPs	Head	Attention	AP (%)
MobileNetv3 × 1.0	5.4 M	220 M	RetinaNet	w/o.	51.2
ResNet50	25 M	3.8 G			59.7
ResNet101	46.3 M	7.6 G			64.8
MobileNetv3 × 1.0	5.9 M	231 M	RetinaNet	w/.	54.1
ResNet50	25 M	3.8 G			62.5
ResNet101	46.3 M	7.6 G			66.3

**Table 5:** Classification results with other methods on the BCTSDB dataset.

Model	Backbone	Head	Params. (M)	FLOPs (G)	Top-1 Acc. (%)	Top-5 Acc. (%)
RetinaNet [15]	ResNet50	Anchor-based	37.74	95.66	96.8	98.9
FCOS [16]	ResNet50	Anchor-free	31.84	78.67	98.2	99.1
Ours.	ResNet50	Anchor-free	37.31	89.95	98.7	99.5

**Table 6:** Comparison of results with other methods on the BCTSDB dataset.

Model	Backbone	Head	AP	AP_50_	AP_75_	AP_S_	AP_M_	AP_L_	FPS
Faster R-CNN [10]	ResNet50	Anchor-based	70.2	94.7	86.0	65.3	76.5	84.5	28
Cascade R-CNN [11]	ResNet50	Anchor-based	75.8	96.7	92.5	72.9	79.3	89.2	23
YOLOv3 [14]	Darknet53	Anchor-based	59.5	92.7	70.4	54.2	70.1	83.8	56
RetinaNet [15]	ResNet50	Anchor-based	59.7	89.4	71.2	47.2	72.5	83.3	52
FCOS [16]	ResNet50	Anchor-free	68.6	95.8	83.9	62.7	75.7	83.9	61
Ours.	ResNet50	Anchor-free	76.1	97.6	91.4	63.1	77.4	84.5	60

**Table 7:** Comparison results for detection methods on the KITTI dataset.

Methods	Car			Pedestrian			Cyclist			Time (ms)
	Easy (%)	Moderate (%)	Hard (%)	Easy (%)	Moderate (%)	Hard (%)	Easy (%)	Moderate (%)	Hard (%)	
Regionlets [49]	84.75	76.45	59.70	73.14	61.15	55.21	70.41	58.72	51.83	-
Faster R-CNN [10]	87.97	79.11	70.62	78.97	65.24	60.09	71.40	61.86	53.97	142
Mono3D [50]	84.52	89.37	79.15	80.30	67.29	62.23	77.19	65.15	57.88	-
MS-CNN [51]	93.87	88.68	76.11	85.71	74.89	68.99	84.88	75.30	65.27	-
SSD [52]	87.34	87.74	77.27	50.38	48.41	43.46	48.25	52.31	52.13	30
ASSD [53]	89.28	89.95	82.11	69.07	62.49	60.18	75.23	76.16	72.83	30
RFBNet [54]	87.31	87.27	84.44	66.16	61.77	58.04	74.89	72.05	71.01	23
Ours.	90.48	88.03	81.25	83.32	79.35	75.67	85.04	82.33	77.76	22

## Data Availability

Not applicable.

## Abbreviations

AP	Averaged AP at IoUs from 0.5 to 0.95 with an interval of 0.05
AP50	AP at IoU threshold 0.5
AP75	AP at IoU threshold 0.75
APL	AP for objects of large scales (area > 962)
APM	AP for objects of medium scales (322 < area < 962)
APS	AP for objects of small scales (area < 322)
BCTSDB	BUU Chinese Traffic Sign Detection Benchmark
CNN	Convolutional Neural Network
FLOPs	Floating-point operations per second
FPN	Feature pyramid network
FPS	Frames Per Second
GEE	Global Extract Encoder
HOG	Histogram of Oriented Gradients
IoU	Intersection over union
LSTM	Long Short-Term Memory
NMS	Non-Maximum Suppression
RNN	Recurrent Neural Network
SOTA	State-of-the-art
SSD	Single Shot MultiBox Detector
SVM	Support Vector Machine
VRAM	Video random access memory
YOLO	You Only Look Once
